# Salivary Proteome Insights: Evaluation of Saliva Preparation Methods in Mucopolysaccharidoses Research

**DOI:** 10.3390/biomedicines13030662

**Published:** 2025-03-07

**Authors:** Maria-Andreea Soporan, Ioana-Ecaterina Pralea, Maria Iacobescu, Radu Cristian Moldovan, Camelia Alkhzouz, Diana Miclea, Cristina-Adela Iuga

**Affiliations:** 1 Department of Pharmaceutical Analysis, Faculty of Pharmacy, “Iuliu Hațieganu” University of Medicine and Pharmacy, Louis Pasteur Street 6, 400349 Cluj-Napoca, Romania; andreea.ungur@medfuture.ro; 2 Personalized Medicine and Rare Diseases Department, MEDFUTURE—Institute for Biomedical Research, “Iuliu Hațieganu” University of Medicine and Pharmacy, Louis Pasteur Street 6, 400349 Cluj-Napoca, Romania; ioana.pralea@medfuture.ro (I.-E.P.); maria.iacobescu@medfuture.ro (M.I.); moldovan.radu@umfcluj.ro (R.C.M.); 3 Department Mother and Child, Faculty of Medicine, “Iuliu Hațieganu” University of Medicine and Pharmacy, Calea Moților, No. 68, 400370 Cluj-Napoca, Romania; alkhzouz@yahoo.com (C.A.); miclea12diana@gmail.com (D.M.); 4 Medical Genetics Department, Clinical Emergency Hospital for Children, Calea Moților, No. 68, 400370 Cluj-Napoca, Romania

**Keywords:** saliva sample preparation, bottom-up proteomics, in-solution digestion, single-pot solid-phase-enhanced sample preparation (SP3), mucopolysaccharidosis

## Abstract

**Background:** This research aimed to compare the traditional in-solution digestion (inSol) and solid-phase-enhanced sample preparation (SP3) methods for salivary proteomics, with a focus on identifying mucopolysaccharidosis (MPS)-relevant proteins. **Methods:** Saliva samples were processed under multiple analytical conditions, including two precipitation methods (methanol or incubation with trichloroacetic acid), paired with either Rapigest or 8M urea/2M thiourea (UT) solubilization buffers. Additionally, the SP3 method was directly applied to raw saliva without pre-processing. Proteome coverage, reproducibility, digestion efficiency, and gene function were assessed. **Results:** The inSol method consistently provided superior proteome coverage, with trichloroacetic acid precipitation and Rapigest buffer yielding 74 MPS-relevant proteins, compared to 40 with SP3 MeOH UT. Both methods showed high digestion efficiency, particularly with Rapigest buffer, achieving over 80% full cleavage across conditions. Functional analysis revealed broad similarities, with protocol-specific impacts on protein classes and cellular components. **Conclusions:** This study is the first to compare SP3 and in-solution digestion for salivary proteomics, emphasizing the importance of method selection to address matrix-specific challenges. The results highlight the robustness of inSol for comprehensive proteome profiling and SP3′s potential for streamlined clinical workflows, offering valuable insights into optimizing salivary proteomics for biomarker discovery in MPS and other diseases.

## 1. Introduction

Saliva, a complex biological fluid, has emerged as a valuable matrix for biomarker discovery due to its non-invasive collection, ease of handling, and ability to reflect both local and systemic pathophysiological states. The salivary proteome comprises thousands of proteins, primarily secreted by the acinar cells of the salivary glands, with additional contributions from plasma via transcellular transport. Despite its potential as a diagnostic tool, the lower concentrations of many biomolecules (including proteins) in saliva compared to those in blood present challenges for its use in clinical applications [1]. However, its simple, non-invasive collection process, particularly beneficial in vulnerable populations such as neonates and children, positions saliva as an attractive alternative to blood for proteomic studies [2]. Given these advantages, optimizing analytical methods for salivary proteomics is crucial to fully assess its potential.

A clinical area where salivary proteomics holds promises is the study of mucopolysaccharidoses (MPSs), a group of rare, chronic, systemic lysosomal storage disorders caused by deficiencies in enzymes responsible for glycosaminoglycan (GAG) degradation. While urinary GAGs have long been used as primary biomarkers, their limitations such as weak correlation with clinical severity and variable response to therapy highlight the need for additional markers. Among others, proteins can serve as valuable indirect biomarkers, reflecting disease pathophysiology beyond GAG accumulation. Molecules like fibroblast growth factor-2 (FGF-2), heparin cofactor-II-thrombin complex (HCII-T), and dipeptidyl peptidase IV (CD26) provide insights into secondary cellular dysfunction, inflammation, and therapeutic responses. Thus, proteomic approaches can identify novel biomarkers that better correlate with disease burden and treatment outcomes [3]. Notably, hypersalivation (sialorrhea) is a common feature of MPS patients, providing an accessible biological fluid for studying disease-specific biomarkers. In children with MPS, intellectual disability is one of the most common causes of sialorrhea, with additional causes including macroglossia, respiratory obstruction, and neurological impairment [4,5]. The prevalence of hypersalivation in MPS patients and the ease of saliva collection supports the use of this biological fluid in proteomic studies aimed at discovering biomarkers relevant to this rare disorder. However, to achieve reliable and reproducible results, efficient sample preparation techniques must be evaluated and further implemented.

Over time, a variety of sample preparation methods for mass spectrometry (MS)-based salivary proteomics have been introduced, transitioning from traditional techniques like two-dimensional gel electrophoresis (2D-PAGE) [6,7] to more advanced and efficient protocols like filter-aided sample preparation (FASP) [8,9]. The 2D-PAGE method is used for its ability to identify protein isoforms and post-translational modifications (PTMs), but recently it is less commonly used due to its slow and labor-intensive nature. In contrast, in-solution digestion, which allows for the simultaneous proteolysis of multiple proteins, has been widely used in salivary proteomics [6,8,9,10,11,12,13]. While this method is efficient, it also has certain limitations, particularly in identifying transmembrane proteins or proteins with extensive post-translational modifications, and it often requires subsequent desalting and concentration steps. More recently, the FASP protocol has emerged as a method that uses filtration to remove detergents that could interfere with trypsin digestion [8,9]. FASP employs ultrafiltration spin devices with molecular weight cut-off membranes to facilitate protein solubilization, denaturation with urea, and optimized proteolysis. Although these newer approaches improve workflow efficiency, challenges such as incomplete protein recovery and the need for downstream purification persist, highlighting the need for further advancements in sample preparation techniques for salivary proteomics.

Advances in salivary proteomics have facilitated the identification of biomarkers for a wide range of diseases, from Sjögren’s syndrome [14] to cancer [15]. Despite these advancements, the lack of standardized sample preparation methods remains a critical barrier in achieving consistent and reliable proteomic analyses. Efficient and reproducible sample preparation is essential for generating high-quality data in MS-based proteomics. In this regard, bottom-up proteomics offers a powerful strategy, where precise and controlled steps—such as protein extraction, denaturation, proteolytic digestion, and the removal of interfering substances like detergents and salts—are pivotal to unlock the full potential of MS-based analyses [16].

Within this framework, two key approaches have gained prominence: in-solution digestion and solid-phase-enhanced sample preparation (SP3). In-solution digestion relies on chemical and thermal denaturation followed by trypsin digestion and has proven effective for various proteins and biological fluids, though it often requires additional steps for desalting and concentration [9]. Recently, solid-phase-enhanced sample preparation (SP3) was developed as a promising approach for the analysis of saliva samples. The SP3 method employs paramagnetic beads to process proteins quickly and efficiently while minimizing sample losses. SP3 facilitates the removal of interfering substances and supports the analysis of complex protein mixtures, making it a promising alternative to traditional in-solution approaches. The SP3 method was developed to improve upon traditional in-solution digestion methods by minimizing sample loss, reducing contamination, and increasing reproducibility. Briefly, the protocol uses paramagnetic beads coated with a functionalized surface, allowing for the isolation of proteins from complex biological matrices. The protocol allows for easy protein purification and enhanced direct, on-bead trypsin digestion, minimizing interference with the digestion step, which is a common issue with traditional in-solution digestion. Overall, SP3 significantly speeds up the sample preparation process by combining multiple steps into one streamlined procedure [17].

Despite the growing use of these methods, a systematic comparison of their efficacy in salivary proteomics has not been fully explored. Therefore, the aim of this study was to evaluate and compare the performance of in-solution digestion and SP3, with a focus on identifying proteins relevant to MPS. By addressing the inconsistencies in saliva sample preparation, this study advances the field of salivary proteomics and enhances its potential for biomarker discovery in MPS.

## 2. Materials and Methods

### 2.1. Saliva Collection

Unstimulated whole saliva samples were collected from 10 healthy volunteers (4 males, 6 females; aged 25–46) following the protocol described by Laputková et al. [18]. Briefly, the volunteers were asked to spit into a sterile 50 mL Eppendorf tube for over 10–15 min. To avoid interferences with circadian rhythm, samples were collected between 9 and 11 a.m., and participants were asked to refrain from eating and drinking for one hour before collection [19]. Approximately 5 mL of saliva was obtained and immediately centrifuged at 9500 rpm for 15 min at 4 °C to remove cell debris. The supernatant of each sample was aspirated and combined to form a pooled saliva sample. Biological replicates of this sample were stored at −80 °C until further analysis as 100 µL aliquots.

### 2.2. Protein Extraction

Two precipitation agents and two buffers were evaluated for protein retrieval from pooled saliva samples. These served as pre-processing steps before applying the methods compared (inSol and SP3) and are treated as conditions in this manuscript. Saliva aliquots of 100 µL were thawed on ice and subjected to protein precipitation using either methanol (MeOH) or incubation with trichloroacetic acid for 60 min (TCA60). Protein pellets were dissolved in either 100 µL of 8M urea/2M thiourea (UT) or 0.1% Rapigest^®^ (Waters Corporation, Milford, MA, USA) prepared in 50 mM ammonium bicarbonate (R). Protein extraction was enhanced by sonication treatment for 3 × 3 s at 50% power (Bandelin Electronic GmbH, Berlin, Germany). After centrifugation at 15.000× *g* for 15 min at 4 °C, the supernatants were transferred to new low protein binding tubes. Total protein concentrations were measured using the microBradford assay (BioRad Laboratories, Munich, Germany) [20] against a standard curve of bovine serum albumin (BSA).

### 2.3. Sample Preparation for LC-MS Analysis

**Figure 1:**
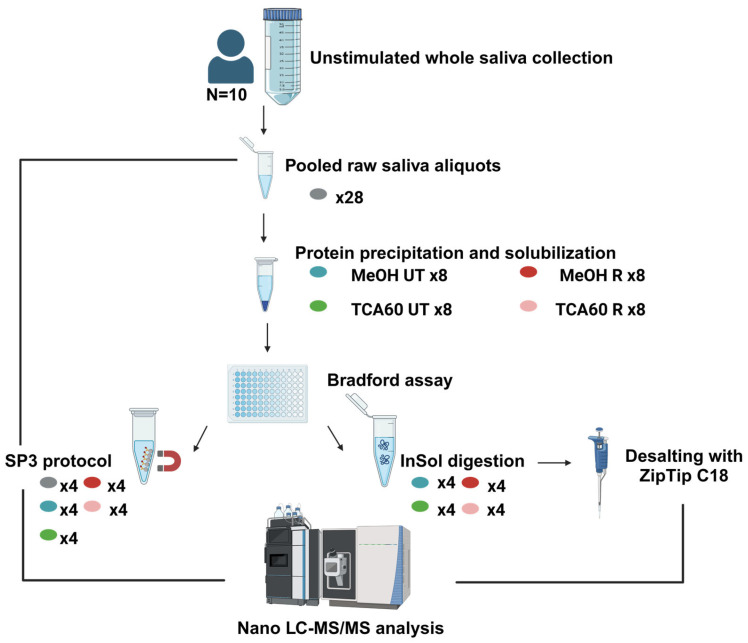
A schematic workflow of the experimental design (Created in https://BioRender.com).

Two different sample preparation protocols for mass spectrometry analysis were applied in this study: the traditional in-solution procedure (inSol) and the more recently developed SP3 protocol. Four biological replicates, each consisting of 100 µL of pooled saliva, were included for each condition tested. The SP3 protocol was additionally applied directly on raw saliva pools (SP3 direct). A summary of the study design is provided in Figure 1.

#### 2.3.1. In-Solution Method (inSol)

A sample volume corresponding to 2 µg of proteins was subjected to reduction with 25 mM dithiothreitol (DTT) at 60 °C for one hour, followed by alkylation with iodoacetamide (IAA) at a final concentration of 40 mM for 30 min at 37 °C. Proteolytic cleavage was performed overnight at 37 °C using proteomics-grade trypsin (MilliporeSigma, Burlington, MA, USA) at a 1:50 enzyme-to-protein ratio. The digestion was quenched with 5% acetic acid. Samples with Rapigest^®^ were further centrifuged at 13,000 rpm for 10 min, and the supernatant was transferred into a new low protein binding tube. All samples were subjected to purification using ZipTip μC18 (Millipore-Sigma, Burlington, MA, USA) according to the manufacturer’s protocol. Eluted peptides were then concentrated by evaporation using a vacuum concentrator (Thermo Fisher Scientific, Waltham, MA, USA) and subsequently re-solubilized in 20 µL of 0.1% formic acid.

#### 2.3.2. Single-Pot, Solid-Phase-Enhanced Sample Preparation (SP3)

For the SP3 protocol, reduction and alkylation were performed prior to protein digestion. Briefly, samples (2 µg of protein) obtained with each precipitation method and buffer were reduced with DTT (to end concentration of 2.5 mM, 30 min at 37 °C) and alkylated with IAA (final concentration 10 mM, 15 min at 37 °C in the dark). Further, the SP3 protocol developed by Hughes et al. [17] was applied with some modifications. Magnetic beads (Sera-MagTM Carboxylate-Modified Magnetic SpeedBeads, CAT#45152105050250 and CAT#65152105050250, Cytiva, Marlborough, MA, USA) were combined at a 1:1 ratio (*v*/*v*, in HPLC grade water) for a final concentration of 20 µg/µL. The obtained bead suspension was added at a 2:1 *v*/*v* ratio (beads to proteins) to each sample. Protein binding on the magnetic beads was induced by the addition of 100% acetonitrile (ACN). After 18 min incubation at RT, the beads were immobilized using a magnetic rack, and the supernatant was discarded. Beads were washed twice with 70% ethanol, followed by two washing steps with 100% ACN. After removing the supernatant, the beads were dried under the fume hood for 5–10 min. On-bead digestion of proteins was then performed overnight at 37 °C using proteomics-grade trypsin (1:50 enzyme-to-protein ratio) and 20 mM ammonium bicarbonate (ABC). To quench digestion and enhance peptide binding to the magnetic beads, 100% ACN was introduced. Samples were incubated and subjected to the washing step, as described above. Finally, peptides were eluted from the beads by dimethyl sulfoxide (DMSO 2% *v*/*v* in water). The supernatant was transferred into injection vials and diluted 1:2 with 0.2% formic acid. Samples were kept at −80 °C until MS analysis.

#### 2.3.3. Single-Pot, Solid-Phase-Enhanced Sample Preparation Using Raw Saliva (SP3 Direct)

The SP3 direct protocol was performed as outlined previously (see SP3 protocol in Section 2.3.2) with the key modification of its direct application to raw pooled saliva samples, without the pre-processing steps of precipitation and re-solubilization of the protein pellet in a subsequent buffer.

### 2.4. Liquid Chromatography Tandem Mass Spectrometry Analysis (LC-MS/MS)

Label-free LC-MS/MS protein profiling was performed using an Acquity UPLC M-class^®^ system (Waters Corporation, Milford, MA, USA) coupled with a SYNAPT G2-Si High-Definition Mass Spectrometer (Waters Corporation, Wilmslow, UK). Peptides were trapped on Symmetry C18 (180 µm × 20 mm, 5 µm particle size, Waters Corporation, Milford, MA, USA) for 2 min at 5 μL/min in 0.5% solvent B (0.1% (*v*/*v*) formic acid in ACN). Peptides were separated on a reverse-phase HSS C18 column (75 µm × 150 mm, 1.8 µm particle size, Waters Corporation, Wexford, Ireland) at a flow rate of 300 nL/min over a 45 min multistep concave gradient ranging from 5 to 85% solvent B. The analytical column temperature was set to 50 °C. Glu-1-Fibrinopeptide B (100 fmol/μL) was used as a lock-mass compound. For all MS measurements, spectra were collected in resolution positive ion mode over the mass range of 50–2000 m/z with a scan time of 0.5 s and the following settings: source temperature 80 °C, sampling cone voltage of 30 V, desolvation temperature 350 °C, cone gas flow 30 L/h.

Data was obtained using two label-free data-independent acquisition modes, namely, MS^E^ and HDMS^E^. For MS^E^ acquisition, collision energy was ramped from 18 to 40 V for the high-energy scan, while for HDMS^E^ mode, a ramp transfer energy from 19 to 45 V was used. For method and protocol comparisons, pooled saliva samples were run in triplicate with the optimal column load in HDMS^E^ mode.

### 2.5. Data Processing

For the optimal column load, data processing and protein identification was performed using ProteinLynx Global Server (PLGS) version 3.0.3 (Waters Corporation, Milford, MA, USA). For the HDMS^E^ acquisitions, the optimal low-energy (LE) and high-energy (HE) threshold settings were determined using the PLGS threshold inspector (V.2.3, Build 2, Waters Corporation, Milford, MA, USA).

Raw MS data files for the remaining triplicates were processed by Progenesis QIP V.4.2 (Nonlinear Dynamics, Waters Corporation, Milford, MA, USA). Post-acquisition data were lock-mass corrected using the doubly charged monoisotopic ion of Glu-1-Fibrinopeptide B and aligned to the most suitable reference run identified automatically by the software. Normalization was performed using the default “normalize to all proteins option”, and data were searched against a target–decoy Human UniProtKB/Swiss-Prot database containing 20,587 proteins (downloaded August 2023). Search settings included up to one missed cleavage for trypsin digestion, carbamidomethylation of cysteine as a fixed modification, and oxidized methionine as a variable modification. A minimum false discovery rate (FDR) of 1% was allowed, with ion match requirements set as follows: minimum one fragment ion match per peptide ion, three fragment ions matched per protein identification, and at least one peptide match per protein identification. The minimal length required for a peptide was five amino acids, and the MS/MS tolerance was set at 10 ppm. Relative quantitation using non-conflicting data was further performed, and subsequently, the reviewed list of proteins was exported for analysis.

### 2.6. Data Analysis

MetaboAnalyst (Version 6.0) (accessed on 1 October 2024) was used to identify significantly differentially expressed proteins in SP3 and inSol conditions: proteins with more than 30% missing values were removed, and remaining missing values were estimated using k-nearest neighbors based on similar features (KNN feature-wise option). Data were log10 transformed, and for differential expression analysis, a two-sample *t*-test with a *p*-value threshold of 0.05 and a fold change FC ≥ 1.2 was applied. Volcano plot graphical representations were generated within the same tool.

Functional analysis of the proteome data was performed using various tools: SubcellulaRVis was employed for analyzing cellular component gene functions, and the PANTHER Classification System (Version 17.0) [21] was used for gene function analysis, utilizing the PANTHER GO-Slim Biological Process, PANTHER GO-Slim Molecular Function, and PANTHER Protein Class ontologies. Graphical representations of the functional analysis were generated using GraphPad Prism (Version 8.0) and Morpheus [22]. For proteome-level comparisons, Venny (Version 2.1) [23] and Shiny 2 IMetaLab (Version 0.8) [24] were used, while the Human Proteome Atlas (Version 24.0) [25] and Human Salivary Proteome (Version 2.0) [26] served as reference databases.

## 3. Results

Optimizing sample preparation protocols is crucial for establishing methods that are both reproducible and efficient in bottom-up proteomics. In this study, in-solution digestion (inSol) and single-pot solid-phase-enhanced sample preparation (SP3) were chosen to compare different aspects of sample preparation for salivary proteomics.

### 3.1. Optimal Column Load Assessment

To thoroughly evaluate the impact of column loading, we employed both MS^E^ and HDMS^E^ (ion-mobility-enhanced MS^E^) acquisition methods, as they offer distinct advantages and limitations in proteomic analysis. MS^E^ captures information on both precursor and fragment ions in an unbiased manner by alternating MS scans at low and high collision energies. While this method effectively provides full-scan coverage of all ions, it is prone to column overloading, which can lead to fewer protein identifications. In contrast, HDMS^E^ incorporates ion-mobility separation (IMS), adding a drift-time-based separation dimension that aligns precursor and product ions by both retention and drift time, thereby increasing peak capacity and improving proteome coverage up to 60% compared to MS^E^ [27].

In order to determine the required sample quantity for our platform, representative pools of already extracted proteins from one saliva pool corresponding to each condition tested for the inSol and SP3 methods were prepared, respectively, each with two technical replicates per protocol, resulting in a total of four pooled representative samples (inSol_pool1_all conditions, inSol_pool2_all conditions, SP3_pool1_all conditions, and SP3_pool2_all conditions). After tryptic digestion and reconstitution of the samples as mentioned above, equal volume (5 µL) of each of the four biological replicates/method were combined into a representative pool (20 µL) (Figure S1A).

Using both methods, we aimed to evaluate the impact of column load on proteome coverage and to further define the optimal sample quantity for our platform. We analyzed different amounts of peptides ranging from 100 to 400 ng, using a 45 min gradient in both MS^E^ and HDMS^E^. Data processing with PLGS indicated that the number of proteins identified varied between the two acquisition modes, with MS^E^ achieving the highest number of identifications at 300 ng of protein and HDMS^E^ at 400 ng of protein for both protocols (inSol and SP3) (Table S1). The impact of on-column loading amounts on protein and peptide identifications in MS^E^ and HDMS^E^ acquisition modes is illustrated by the number of proteins and peptides detected per each tested loading amount (Figure S1B).

### 3.2. Overall Comparison of In-Solution Digestion and SP3 Protocol for Salivary Proteomics

#### 3.2.1. Proteome Identification and Coverage

**Figure 2:**
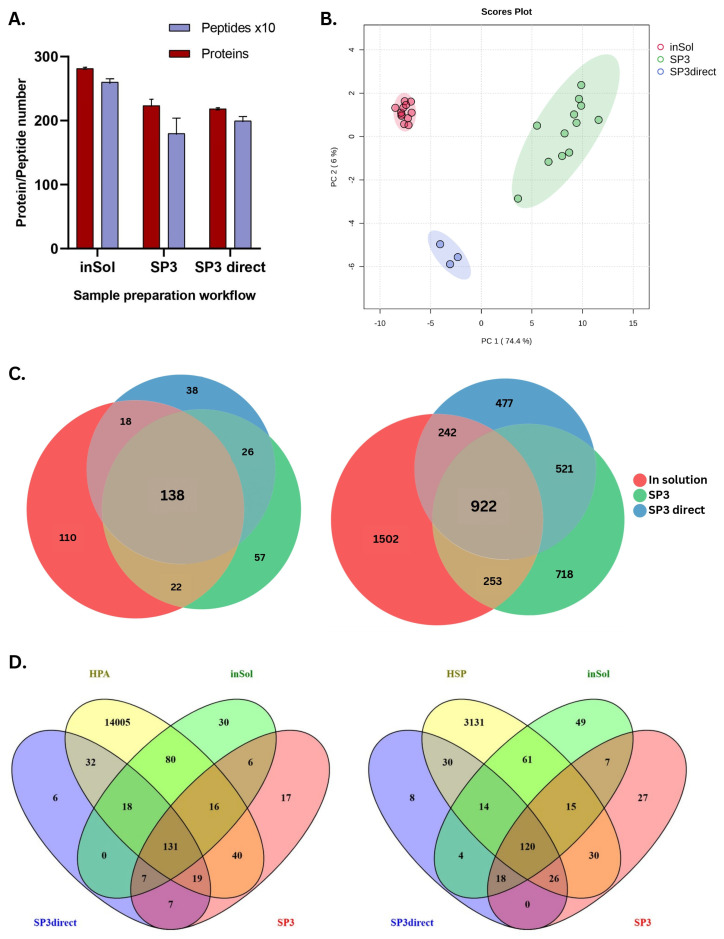
(**A**) Total number of proteins and peptides identified for all replicates in each workflow for all tested conditions. (**B**) Principal component analysis for saliva. (**C**) Area proportional Euler diagrams (Shiny2 iMetaLab) showing the overlap of unique proteins and peptides identified. (**D**) Venn diagrams showing the comparison of proteins identified by experimental methods with two salivary databases: the Human Salivary Proteome (HSP) and Human Proteome Atlas (HPA).

Saliva samples from each experimental procedural condition and method (inSol or SP3) were analyzed in triplicate, with an optimal column load of 400 ng of protein, in HDMS^E^ only. Data were processed using Progenesis QiP across three distinct experimental approaches: inSol, SP3, and SP3 direct, each performed with corresponding triplicates for each condition tested. As expected, different methods resulted in different numbers of protein and peptide identifications, ranging between 2162 and 2919 for peptides and 220 and 288 for proteins (Figure 2A). We observed that the inSol digestion yielded the highest number of peptide (2919) and protein (288) identifications. The SP3 method (2.3.2) also yielded a comparable number of peptides (2414) and proteins (243) to the application of the SP3 method directly to raw saliva (2.3.3), which resulted in 2162 peptides and 220 proteins.

To provide insight into how well the identified proteins reflect distinct proteomic signatures for each method, we performed a principal component analysis (PCA) using MetaboAnalyst (Version 6.0) on all proteins identified across the three methods. The PCA plot (Figure 2B) revealed a clear separation of samples by method, indicating distinct proteome profiles, which highlights the influence of the extraction protocol on the proteome landscape and emphasizes the specificity of each method.

We identified a substantial overlap in saliva protein identifications across different sample preparation workflows, with 138 proteins (33.7% of the total identified proteins) common to the methods applied (Figure 2C). However, the overlap at the peptide level was slightly lower, comprising only 19.9% of the 4635 total peptides. Among the workflows, inSol digestion produced the highest number of unique proteins (110) and peptides (1502).

To ensure the reliability of our findings, we further compared the proteins extracted from saliva to well-established databases, such as the Human Salivary Proteome (HSP) [26] and Human Proteome Atlas (HPA) (proteinatlas.org—accessed on: 2 September 2024) [25], (Figure 2D). Both the HSP and HPA databases were used due to their differences in terms of data source, tissue specificity, detection techniques, and biological considerations. All methods resulted in >70% overlap of the total proteins identified compared with HSP and HPA. For SP3 using raw saliva (Section 2.3.3), we were able to identify the highest percentage of common proteins with the databases of interest (91% for HPA, and 86% for HSP). The inSol method (Section 2.3.1) and SP3 (Section 2.3.2) had similar coverages for HPA (88% and 85%) and HSP (73% and 79%), respectively (Figure S2).

#### 3.2.2. Comparative Functional Analysis of Protein Profiles Across Proteomic Methods

Gaining more insight into the molecular processes in which identified proteins are involved or play functional roles is fundamental for unraveling the mechanisms underlying various pathologies. Functional analyses, such as cellular localization, molecular functions, biological processes, and protein classes, provide valuable insights into the biological contexts of identified proteins and the pathways they influence. Comparing results from different proteomics methods, such as inSol digestion and SP3, enhances the reliability of functional annotations and ensures a more robust understanding of the molecular processes revealed by proteomics-based approaches.

Cellular component analysis of the inSol and SP3 proteomes revealed a similar distribution of extracted proteins in cellular compartments, with most proteins associated with the extracellular region, cytoplasm, nucleus, and intracellular vesicle (Figure S3A). However, a slight difference was observed with the inSol method, which identified fewer cytoskeletal proteins compared to the other methods. Despite this, inSol demonstrated the highest mapping efficiency, with 87.25% of the identified proteins successfully mapped to cellular components, exceeding the coverage achieved by SP3 (85.18%) and SP3 direct (85.9%), which could suggest that while all methods broadly captured similar cellular components, inSol may provide an enhanced coverage of the proteome.

From the biological process perspective, most proteins are predominantly involved in cellular processes, responses to stimuli, or metabolic processes. The SP3 protocol extracted a greater number of proteins associated with developmental, reproductive, or growth processes compared to inSol digestion. In the category of molecular functions, proteins with binding and catalytic activity were among the most represented in the tested methods, followed by proteins with structural molecule activity and cytoskeletal motor activity for the SP3 protocol. Regarding protein class, the inSol protocol identified a higher proportion of proteins associated with defense/immunity while showing a lower percentage of cytoskeletal proteins and those involved in metabolite interconversion compared to the SP3 protocol. The remaining protein classes were similarly represented across the tested methods (Figure S3B).

For the inSol method, the functional analysis of cellular components revealed no significant differences between the tested conditions (Figure S4A). Most proteins were localized to the extracellular region, cytoplasm, intracellular vesicles, or nucleus. The percentage of mapped genes for protein identification was similar across conditions. These observations emphasize that the inSol method is robust and reproducible regardless of the applied condition. For the SP3 method (Figure S4B), cellular components analysis showed a similar pattern across the tested conditions, as observed for the inSol method. However, it is noteworthy that more cytoskeletal proteins were extracted when using Rapigest compared to UT.

Gene functional analysis performed with the PANTHER Classification System (Version 17.0) revealed similarity in the extracted proteins across the tested conditions for both the inSol and SP3 methods (Figure S5). For the inSol method, using UT buffer results in a slight increase in proteins involved in metabolic processes, while MeOH precipitation yields more proteins related to metabolite interconversion. In the SP3 method, TCA precipitation leads to the extraction of more cytoskeletal proteins and binding activity modulators. Furthermore, applying the SP3 method directly to raw saliva produces results comparable to those obtained under standard extraction conditions.

#### 3.2.3. Reproducibility and Digestion Efficiency Across Experimental Conditions

We evaluated the reproducibility of protein identification across all conditions applied to the inSol and SP3 methods. Our data show that within each condition tested, the reproducibility of protein and peptide identification is more consistent across the technical replicates for the inSol method. The coefficient of variation (CV) for protein intensities showed varying results across conditions, with the highest reproducibility in quantification observed for the inSol method, where 63 proteins (80.8%) and 202 peptides (47.2%) exhibited CV values below 20% for the MeOH R condition. For the SP3 method, the reproducibility was visibly lower (Table S5).

**Figure 3:**
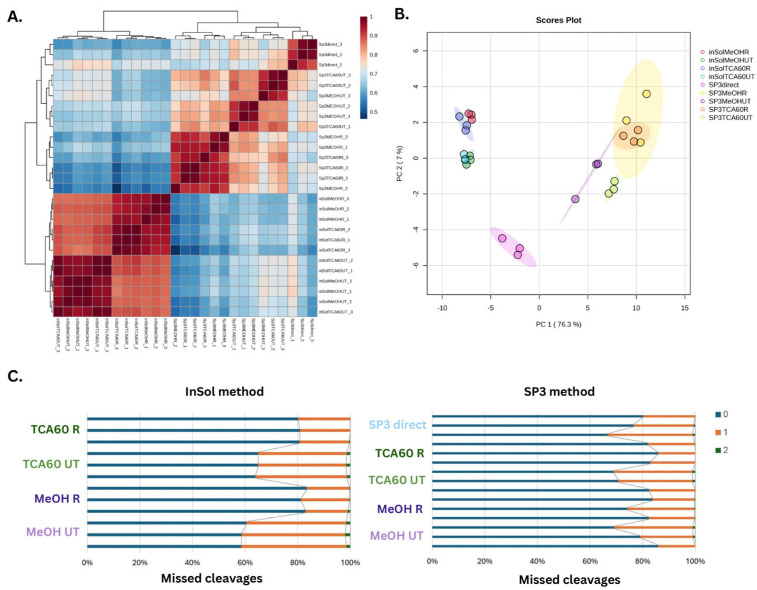
(**A**) Pearson correlation coefficient for log-10 transformed peptide intensity values within three replicates and between different conditions. (**B**) Principal component analysis for log−10 transformed peptide intensity for all saliva samples. (**C**) Percentage of peptides containing zero, one, or two missed tryptic cleavages for three replicates in each workflow.

In a subsequent analysis, we assessed the correlation within and between proteomics samples by calculating the Pearson correlation coefficient for log10-transformed peptide intensity values. The results are visualized in Figure 3A. The intra-sample Pearson correlation coefficients exceeded 0.95 for all inSol conditions and SP3 TCA60 R, demonstrating high technical reproducibility within these sample preparation workflows. In contrast, the inter-sample Pearson correlation values range from 0.5 to 0.8, indicating that while some degree of similarity exists between samples, protocol-specific effects induce variability. Based on correlation analysis combined with principal component analysis (PCA) (Figure 3B), we observed that the inSol protocol exhibits tighter clustering, suggesting greater reproducibility and consistency compared with SP3. Within the inSol method, samples cluster more distinctly based on the buffer used (R/UT), while for the SP3 method, the sensitivity to the buffer used is less pronounced.

Regarding digestion efficiency, our data indicated that Rapigest buffer achieved the highest performance, with an average full cleavage of 83.6% for the SP3 method and 82.5% for the inSol method (Figure 3C). The use of Rapigest consistently resulted in a lower number of missed cleavages, regardless of the precipitation method or protocol employed. Furthermore, the TCA60 R condition exhibited the lowest variability among replicates, regardless of the protocol employed, with coefficients of variation (CV) of 0.44 for inSol and 2.37 for SP3 (Table S6).

### 3.3. Evaluation of In-Solution Digestion and SP3 Approaches

#### 3.3.1. Protein and Peptide Identification

**Figure 4:**
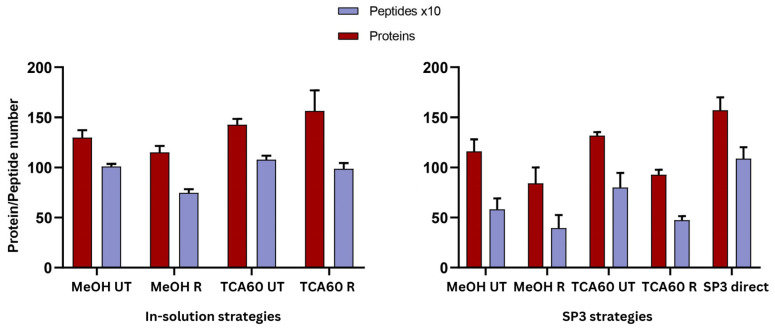
Average number of proteins and peptides identified in each strategy using the inSol and SP3 protocols. Error bars represent the standard deviation from three replicate samples.

For inSol digestion, four different strategies were used, with three replicates per condition. Each replicate was processed individually, and as expected, each condition yielded in slightly different results (Figure 4). The highest number of proteins was observed with TCA precipitation, regardless of the buffer used, with an average of 156 proteins when using Rapigest for solubilization (Table S7).

The comparison of shared proteins across technical replicates within each condition showed an overlap ranging from 64% to 82% (Figure S6). The technical reproducibility of the acquisitions was high, with a coefficient of variation (CV) of 4–13% for proteins and 3–6% for peptides, respectively (Table S7).

For the SP3 protocol, the four strategies mentioned above and the direct application of the SP3 protocol to raw saliva were tested, with three replicates per condition. The highest protein yield was achieved when using raw saliva, resulting in an average of 157 proteins, followed by the TCA60 UT condition, which yielded an average of 132 proteins (Figure 4). Protein overlaps among technical replicates for each condition ranged between 56% and 83% (Figure S6), while technical reproducibility showed coefficients of variation (CV) ranging from 3% to 19% for proteins and 9% to 33% for peptides (Table S7).

#### 3.3.2. Influence of inSol and SP3 Conditions on the Identification of Proteins Relevant to MPS

To determine which of the conditions within the inSol method is more suitable for our purpose, we first evaluated the impact of the solubilization buffer and the precipitation method on the identified proteins (Figure S7). The volcano plots combine the results of fold-change analysis and *t*-tests, enabling the visualization of statistically significant proteins with differential expression under the tested conditions.

For the MeOH procedure, the solubilization buffer does not have a major effect on the number of significantly differentially expressed proteins. However, in the case of TCA precipitation, Rapigest buffer yields 18.7% more proteins compared to UT. Regarding the impact of the precipitation method, TCA results in 12.83% more differentially expressed proteins when using Rapigest buffer, and 33.3% more when using UT, compared to MeOH precipitation.

**Figure 5:**
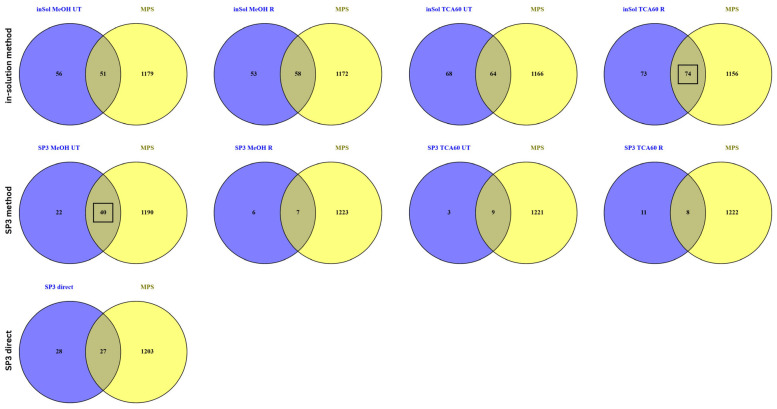
Venn diagrams comparing significantly differentially expressed proteins under tested conditions for the inSol and SP3 methods with the MPS protein reference database.

Proteins with an FC ≥ 1.2 and *p*-value less than 0.05 from each condition were further compared to the reference protein database for MPS (Table S9). Details about the database can be found in Table S8. Within the inSol method, precipitation with TCA followed by solubilization in Rapigest buffer identified the highest number of proteins (74) relevant to MPS (Figure 5).

Within the SP3 method, MeOH precipitation using UT buffer resulted in a 68.25% higher number of significantly differentially expressed proteins compared to Rapigest. Regarding the impact of the precipitation method, when using UT buffer, MeOH precipitation led to a 90% higher number of significantly differentially expressed proteins compared to TCA60. Among the tested conditions, the MeOH UT method yielded the highest number of significantly expressed proteins (40) relevant to MPS. Furthermore, we assessed the impact of the tested conditions within the SP3 protocol in comparison to its direct application to raw saliva. It was observed that MeOH UT and TCA60 R resulted in 63.6% and 14% more proteins, respectively, compared to raw saliva. However, the direct application of the SP3 protocol to raw saliva produced the highest number proteins (27) relevant to MPS. It is noteworthy that the number of significantly expressed proteins with statistical relevance obtained using the SP3 protocol is visibly lower compared to the inSol method, with no statistically significant proteins identified in the SP3_TCA60 UT vs. raw saliva comparison (Figure S7).

## 4. Discussion

The main goal of this study was to compare a traditional in-solution digestion approach with the recently developed solid-phase-enhanced sample preparation (SP3) technology [17,28] for profiling the human salivary proteome, with a focus on identifying proteins relevant to mucopolysaccharidosis (MPS).

In this study, the inSol protocol yielded a slightly higher number of identified peptides and proteins compared to SP3. The inSol method enabled the detection of 288 proteins across all tested conditions, surpassing SP3′s 243 proteins, and 220 proteins when using raw saliva. This superior proteome coverage aligns with the inSol protocol’s robust solubilization and digestion capabilities, particularly when optimized with TCA precipitation and Rapigest buffer. InSol digestion has been widely employed in salivary proteomics, as seen in several studies. However, there are several key methodological differences across these studies that could influence the comparability of our results. These variations include factors such as the processed saliva volume, the choice of precipitation agent, the composition of the solubilization buffer, and, crucially, the type of mass spectrometer employed. For instance, Schwartzova et al. [8] reported one of the highest yields (159 proteins) using an in-solution approach based on acetone precipitation, utilizing amylase-depleted saliva fractions and UT buffer. In contrast, our study, which employed a similar approach on unfractionated saliva—utilizing a precipitation agent and the same solubilization buffer— resulted in the identification of an average of 143 proteins, starting with just 100 µL of sample (TCA60 UT method). Using an approach similar to the one employed in the current study, which utilizes TCA precipitation and UT solubilization, but starting with different sample volumes, Golatowski et al. [10] reported the identification of approximately 160 proteins per subject. This study also emphasized the necessity of standardized saliva processing to limit pre-processing bias. In this context, our study contributes to addressing this gap by evaluating and directly comparing two widely used approaches in proteomics studies. For example, Ventura et al. [29] identified 248 proteins without employing a depletion step and applying a 70 min gradient and an MS^E^ method on a comparable mass spectrometer. Their findings suggest that the depletion of albumin and IgG from saliva samples may not be required. Conversely, a lower yield for in-solution digestion relative to other methods was observed in a study by Zhang et al. [9], which demonstrated that FASP consistently outperformed in-solution digestion in saliva analysis, identifying 488 proteins compared to just 133.

Recently, novel sample processing techniques have been introduced, including single-pot solid-phase-enhanced sample preparation (SP3) [17]. This method was evaluated for its processing efficiency in comparison to FASP using HeLa cell lysate [30] and demonstrated a superior proteomic coverage and more consistent quantification than FASP. However, to date, this technique has not been applied to saliva sample processing. Notably, in our study, SP3 exhibited robust performance in minimizing sample losses, demonstrating good digestion efficiency and effective processing of raw saliva. It achieved a substantial overlap with the HPA (91%) and HSP (86%) databases, along with a high protein yield, averaging 157 protein identifications for raw saliva and 132 for TCA60 UT. These findings underscore the fact that while both methods broadly capture the core salivary proteome, the inSol protocol’s ability to extract a larger number of unique proteins (110) may be advantageous for biomarker discovery in various diseases, including MPS. In contrast, SP3, especially when applied directly to raw saliva, offers a simpler workflow that could be beneficial for clinical settings where time and resources are limited. Beyond saliva, studies using other biological matrices have provided valuable insights into the strengths and weaknesses of various proteomic preparation methods. For instance, Ludwig et al. [31] found that SP3 significantly outperformed FASP and in-solution digestion in terms of proteome coverage and reproducibility when analyzing protein extracts from SW480 colon cancer cell lines. Similarly, Sielaff et al. [30] demonstrated that SP3 provided higher proteome coverage, particularly for low-input samples, compared to FASP and iST. These findings highlight SP3′s advantages in handling complex and dynamic matrices.

Several studies [11,12,13,32] have reported protein identification ranges in saliva samples comparable to our findings. For instance, between 83 and 192 proteins were identified in pregnant women [11], up to 195 proteins in children with autism spectrum disorder [32], and 306 proteins in children with caries using Rapigest as a denaturing buffer [13]. These studies highlight how varying processing approaches, such as buffer choice, can significantly influence protein yields. This variability is consistent with our observation that in-solution digestion is more sensitive to extraction buffer, whereas SP3 exhibits less pronounced clustering based on the buffer used.

Varnavides et al. [33] also emphasized that in-solution digestion is highly sensitive to buffer composition and often requires extensive sample handling, increasing the risk of protein loss and variability. In contrast, SP3′s single-vessel approach minimizes sample loss and maximizes recovery, making it particularly suited for low-abundance proteomes. These observations support our findings that SP3 can provide an efficient and reproducible workflow for saliva proteomics, addressing certain limitations of in-solution digestion.

In our study, the inSol protocol identified a significantly higher number of proteins relevant to MPS compared to SP3. Specifically, TCA precipitation followed by Rapigest solubilization within the inSol protocol yielded 74 MPS-relevant proteins, the highest among all tested conditions. In comparison, the MeOH UT condition in SP3 identified 40 MPS-relevant proteins, while the SP3-direct approach detected 27 such proteins. This indicates that sample preparation and extraction buffer play a critical role in proteome profiling, particularly for rare disease biomarker discovery. As the proteins analyzed originated from the same homogenized biological sample, any differences in quantification cannot be attributed to true biological variation. Rather, the observed up- or down-regulation of proteins reflects biases introduced by the specific bottom-up sample preparation method. Interestingly, despite the SP3 protocol’s ability to process raw saliva effectively, its application in this study identified fewer statistically significantly differentially expressed proteins compared with the inSol method. This may be due to differences in digestion efficiency and reproducibility, as detailed below.

Reproducibility is a critical factor in proteomics, especially for biomarker discovery [31]. In the present study, the inSol protocol demonstrated superior reproducibility across technical replicates, with lower coefficients of variation (CVs) for both proteins and peptides compared to those of SP3. For instance, under the inSol MeOH R condition, 80.8% of proteins exhibited CVs below 20%, indicating high technical consistency. In contrast, SP3 showed higher variability, underscoring the reliability of the inSol protocol for comparative analyses across biological samples. In terms of digestion efficiency, the use of Rapigest buffer emerged as the optimal choice for both protocols, minimizing missed cleavages and achieving a digestion efficiency above 80% in both methods, regardless of the precipitation agent. The TCA60 R condition from the inSol protocol exhibited less variability among replicates and maintained slightly better digestion consistency across conditions.

Both methods revealed a diverse set of proteins involved in critical biological processes, molecular functions, and cellular components. The inSol method identified a higher proportion of proteins related to defense/immunity and extracellular regions, while SP3 showed enhanced identification of proteins involved in developmental and growth processes, potentially broadening its applicability in certain biological contexts and improving our understanding in disease pathophysiology. While the cellular localization of the identified proteins was largely similar across methods, slight differences in protein class distributions reflect the protocol-specific biases introduced during sample preparation. For instance, the SP3 protocol captured more cytoskeletal proteins, while the inSol protocol demonstrated better mapping efficiency to cellular compartments (87.25% vs. 85.18%). Our study highlights that SP3 can be considered a robust alternative to traditional methods, capable of addressing some of these challenges while offering enhanced proteome coverage and reproducibility.

Omics studies on buccal swab [34] and saliva samples [35,36] have demonstrated their potential as rich sources of genomic and proteomic data, offering valuable opportunities to enhance diagnosis and patient care. In the context of MPS I, the detection of α-iduronidase (IDUA) enzyme activity in saliva demonstrates the feasibility of using saliva or buccal swabs for quantifying IDUA levels, reinforcing their promise for non-invasive diagnostic applications. The potential application of salivary proteomics for biomarker discovery in complex diseases like mucopolysaccharidosis (MPS) further underscores its relevance. For instance, Zhang et al. [34] demonstrated the feasibility of employing buccal swabs for MPS diagnostics, emphasizing the need for robust and reproducible sample preparation methods. Our findings suggest that the evaluation of different approaches for salivary proteomics is pivotal to advancing saliva-based diagnostics, particularly for multi-systemic diseases requiring sensitive and efficient proteomic workflows. These findings underscore the broader implications of method-specific biases in proteomics research, where selecting the appropriate protocol is critical not only for maximizing protein identifications but also for ensuring reproducibility and minimizing artifacts.

This study is the first that compares in-solution digestion and SP3 methods in the context of salivary proteomics, providing valuable insights into their applicability for rare disease biomarker discovery. By evaluating these methods with a focus on MPS, we offer a novel perspective on how saliva, a non-invasive biological fluid, can be leveraged for proteomic analysis in rare disease research. However, our study presents certain limitations. Our results were assessed with the purpose of determining which method enhances the detection of proteins that are relevant to MPS, and only two methods were tested. Additionally, the in-house reference protein database used for comparison was created from studies that primarily focused on other biological fluids rather than saliva.

The insights gained from this methodological comparison will serve our group as the foundation for future studies, since we have identified the protocol that maximizes the detection of MPS-relevant proteins. Our next step will be to apply this optimized proteomics workflow to a cohort of MPS patients and further enable the identification of novel salivary biomarkers for this rare disorder.

## 5. Conclusions

To our knowledge, this is the first study that systematically compares SP3 and in-solution digestion methods for salivary proteomics. While SP3 has been successfully applied to other sample types, its use in saliva remains underexplored. By directly comparing SP3 to in-solution digestion, our study addresses a key gap in the literature, providing valuable insights into the strengths and limitations of these protocols and laying the groundwork for future research on salivary proteomics.

Our findings highlight that while both methods identify a comparable number of proteins, in-solution digestion demonstrates superior performance in terms of proteome coverage, reproducibility, and efficiency, making it relevant for biomarker discovery in rare disease research. This analytical comparison provides a foundation for selecting appropriate sample preparation methods to address matrix-specific challenges. Importantly, this study is not a clinical investigation but rather a methodological evaluation that serves as a starting point for future clinical studies focused on saliva-based biomarker discovery.

## Data Availability

Data are contained within the article.

## Supplementary Materials

The following supporting information can be downloaded at: https://www.mdpi.com/article/10.3390/biomedicines13030662/s1, Figure S1: Optimal column load assessment; Figure S2: Venn diagram showing the overlap of protein identifications across the methods compared to salivary databases; Figure S3: Functional analysis using SubCellularRVis and PantherDB; Figure S4: Cellular component gene function analysis using SubCellularRVis for in-solution and SP3 strategies; Figure S5: Gene function analysis using Panther Classification System v.17.0 for in-solution conditions and SP3 conditions; Figure S6: Protein overlap among replicates using different in-solution and SP3 strategies; Figure S7: Volcano plots displaying the statistical *p*-value and the magnitude of abundance changes between each condition for in-solution and SP3 methods; Table S1: Optimal column load assessment; Table S2: Protein list for the in-solution method Progenesis QiP experiment with all conditions; Table S3: Protein list for the SP3 method Progenesis QiP experiment with all conditions; Table S4: Protein list for the SP3 method applied directly to raw saliva Progenesis QiP experiment; Table S5: Number of proteins/peptides with values in all three replicates; Table S6: Digestion efficiency in each condition for all triplicates using inSol and SP3 methods; Table S7: Technical reproducibility analysis of inSol and SP3 strategies for saliva samples; Table S8: Summary of articles included for the construction of the MPS reference protein database; Table S9: MPS protein reference database.
