# Supplementary material for: Salivary Proteome Insights: Evaluation of Saliva Preparation Methods in Mucopolysaccharidoses Research
Source: Biomedicines. 2025 Mar 7;13(3):662. doi: 10.3390/biomedicines13030662 (PMC11940144; doi:10.3390/biomedicines13030662)
Supplement: Supplementary file 1 [file biomedicines-13-00662-s001.zip › Supplementary figures 1_7.pdf]

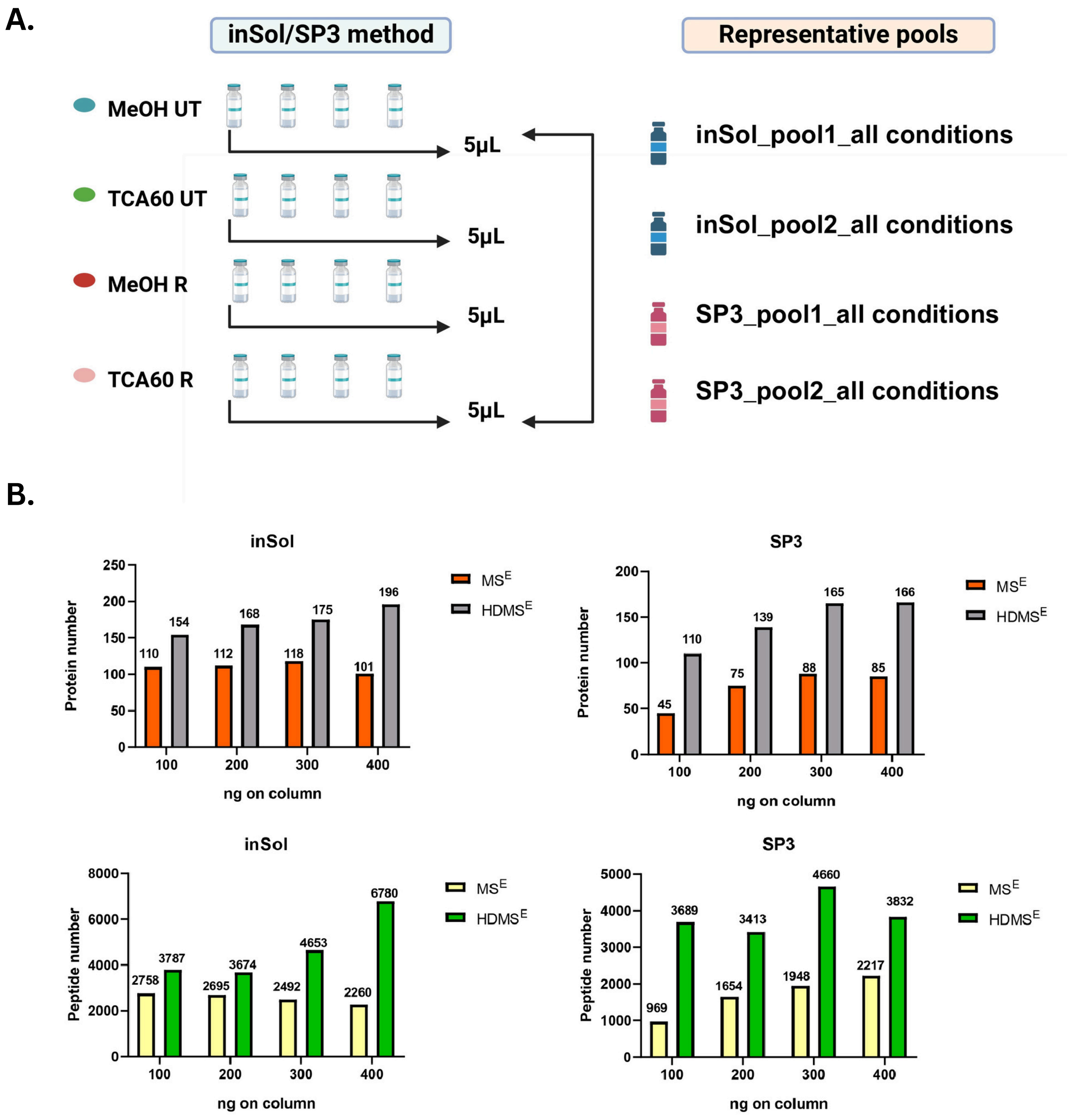

Figure S1: Optimal column load determination. A. Representative sample pools - experimental design B. Protein and peptide number for inSol and SP3 in MSE and HDMSE.

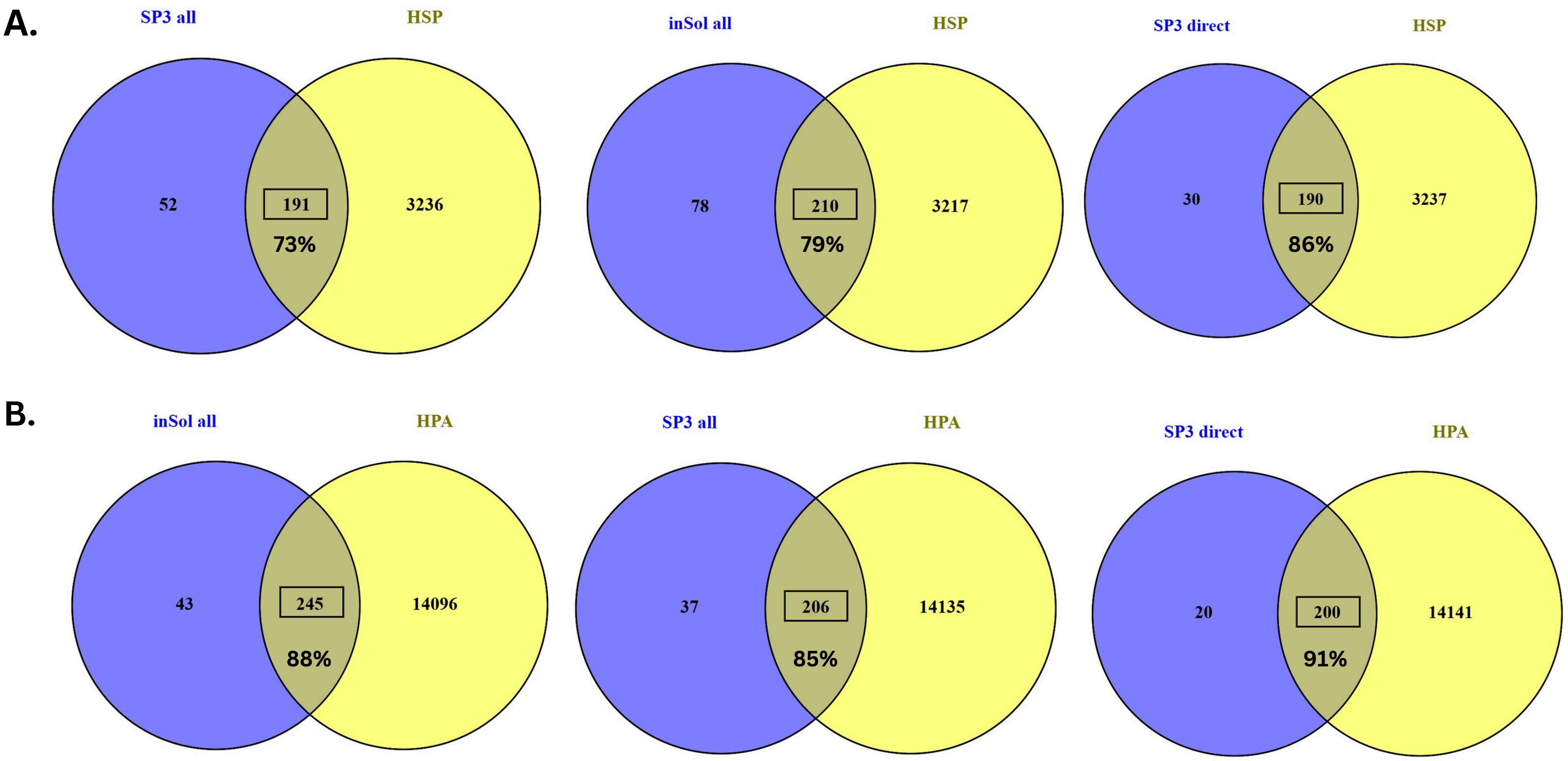

Figure S2: Venn diagram showing the overlap of protein identifications across the methods compared to salivary databases. A. Human Salivary Proteome; B. Human Protein Atlas.

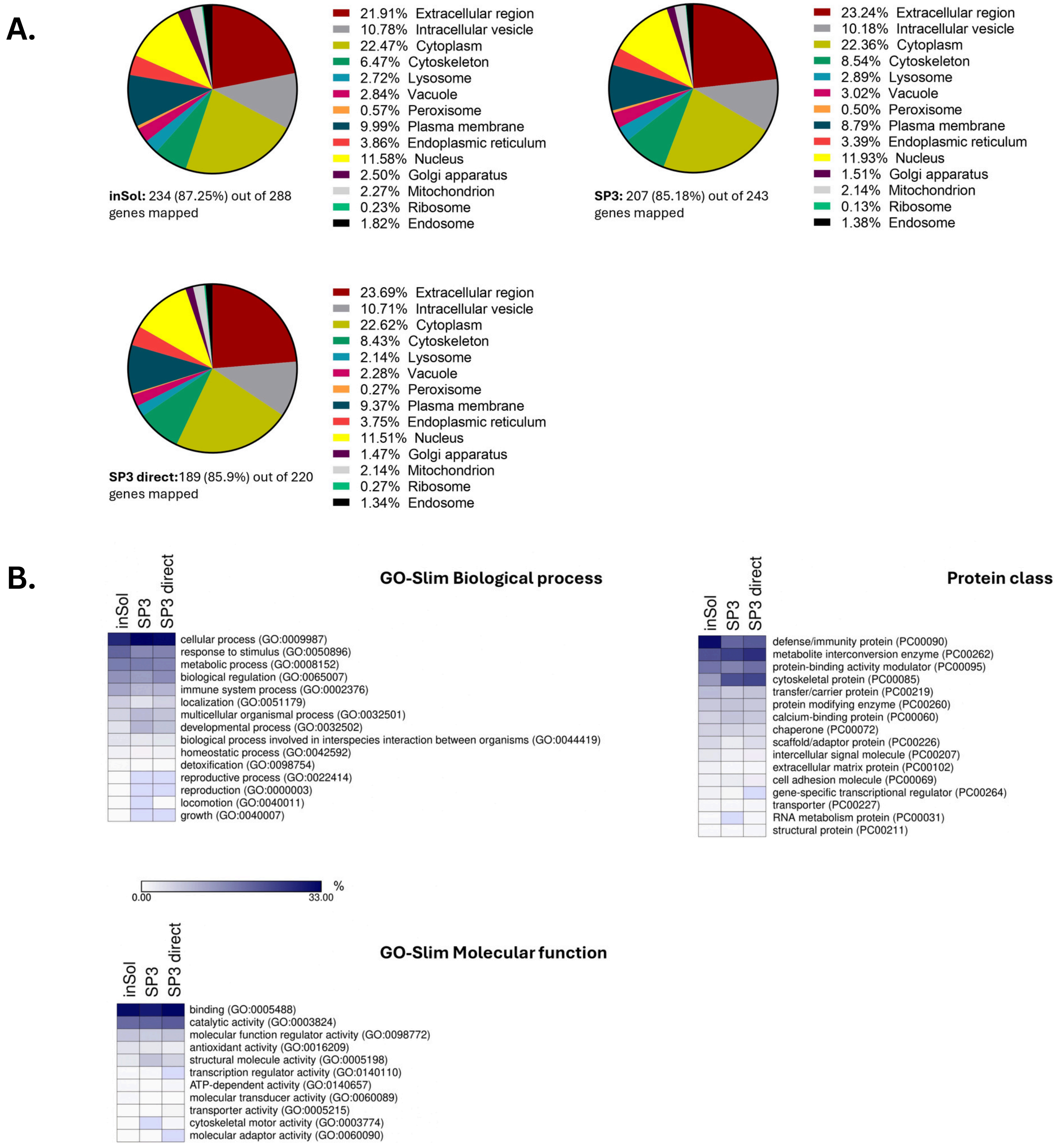

Figure S3: Functional analysis using SubCellularRVis and PantherDB. A. Cellular component gene function analysis for tested methods using SubCellularRVis; B. Gene function analysis using Panther Classification system v.17.0 for tested methods.

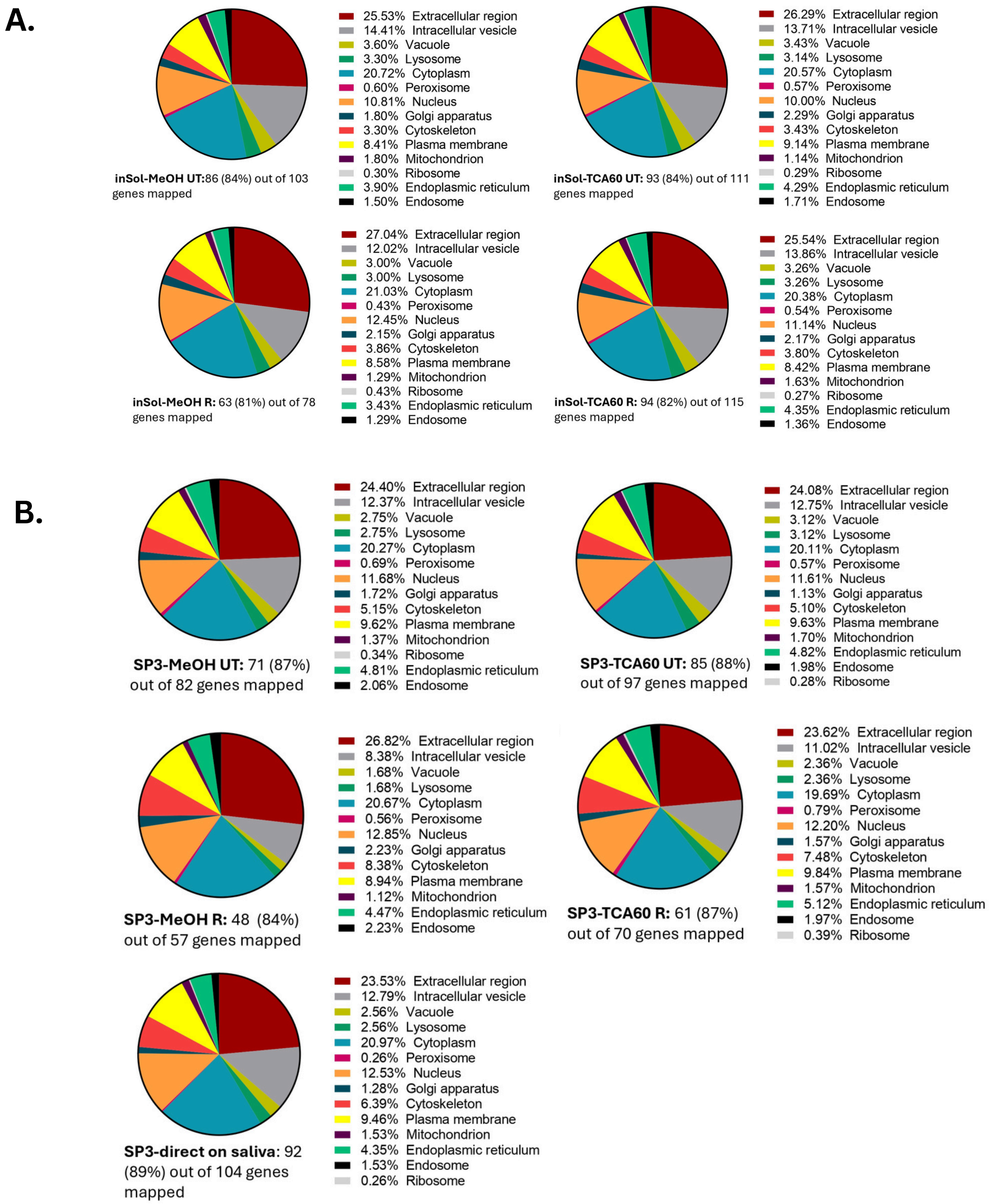

Figure S4: Cellular component gene function analysis using SubCellularRVis for A. in-solution and B. SP3 conditions. Graphical representations were obtained using GraphPad Prism v.8.

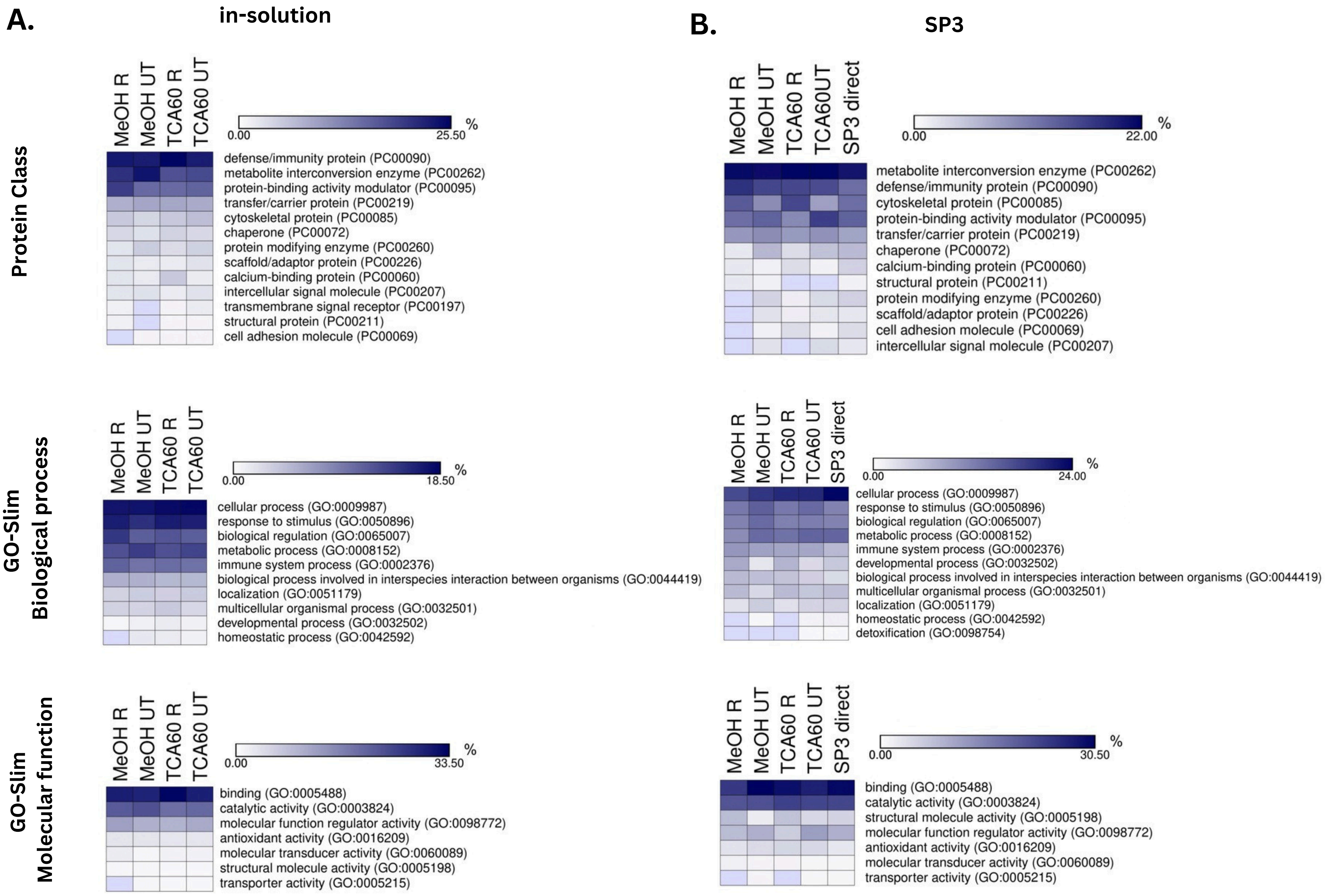

Figure S5: Gene function analysis using PantherDB for A. in-solution and B. SP3 strategies. Graphical representations were obtained using Morpheus.

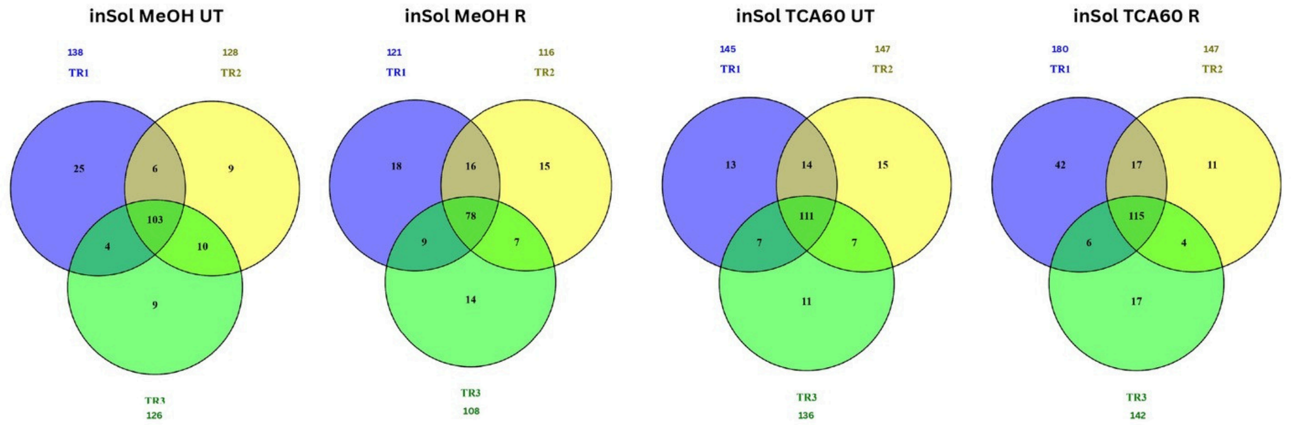

% of common proteins in		
TR1	TR2	TR3
75	80	82

% of common proteins in		
TR1	TR2	TR3
65	67	72

% of common proteins in		
TR1	TR2	TR3
77	76	82

% of common proteins in		
TR1	TR2	TR3
64	78	81

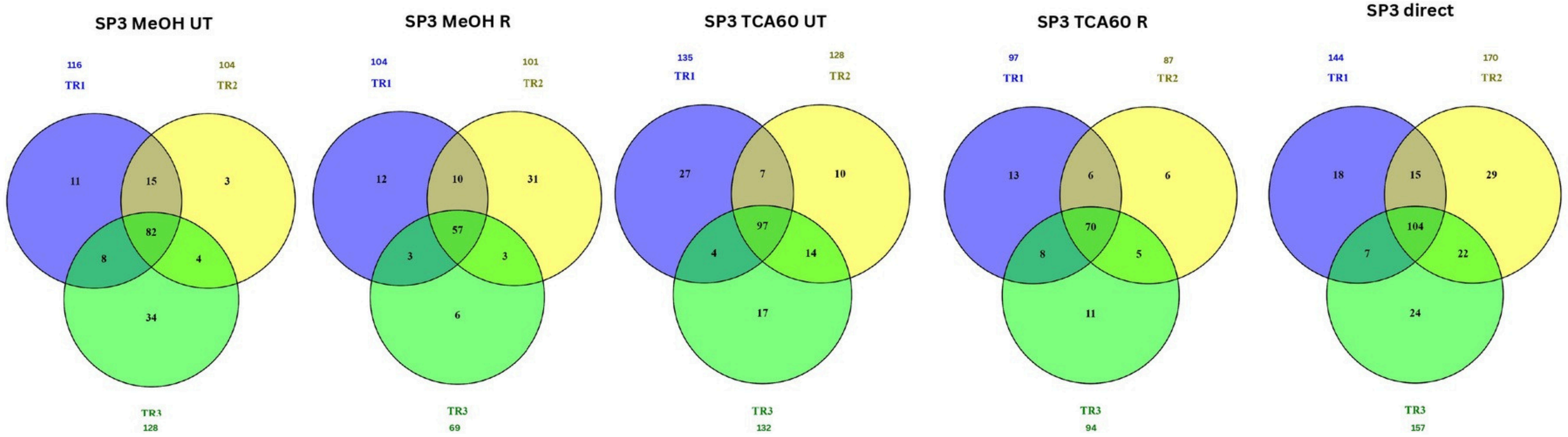

% of common proteins in		
TR1	TR2	TR3
71	79	64

% of common proteins in		
TR1	TR2	TR3
70	56	83

% of common proteins in		
TR1	TR2	TR3
72	76	73

% of common proteins in		
TR1	TR2	TR3
72	80	74

% of common proteins in		
TR1	TR2	TR3
72	61	66

Figure S6: Protein overlap among replicates using different in-solution and SP3 strategies.

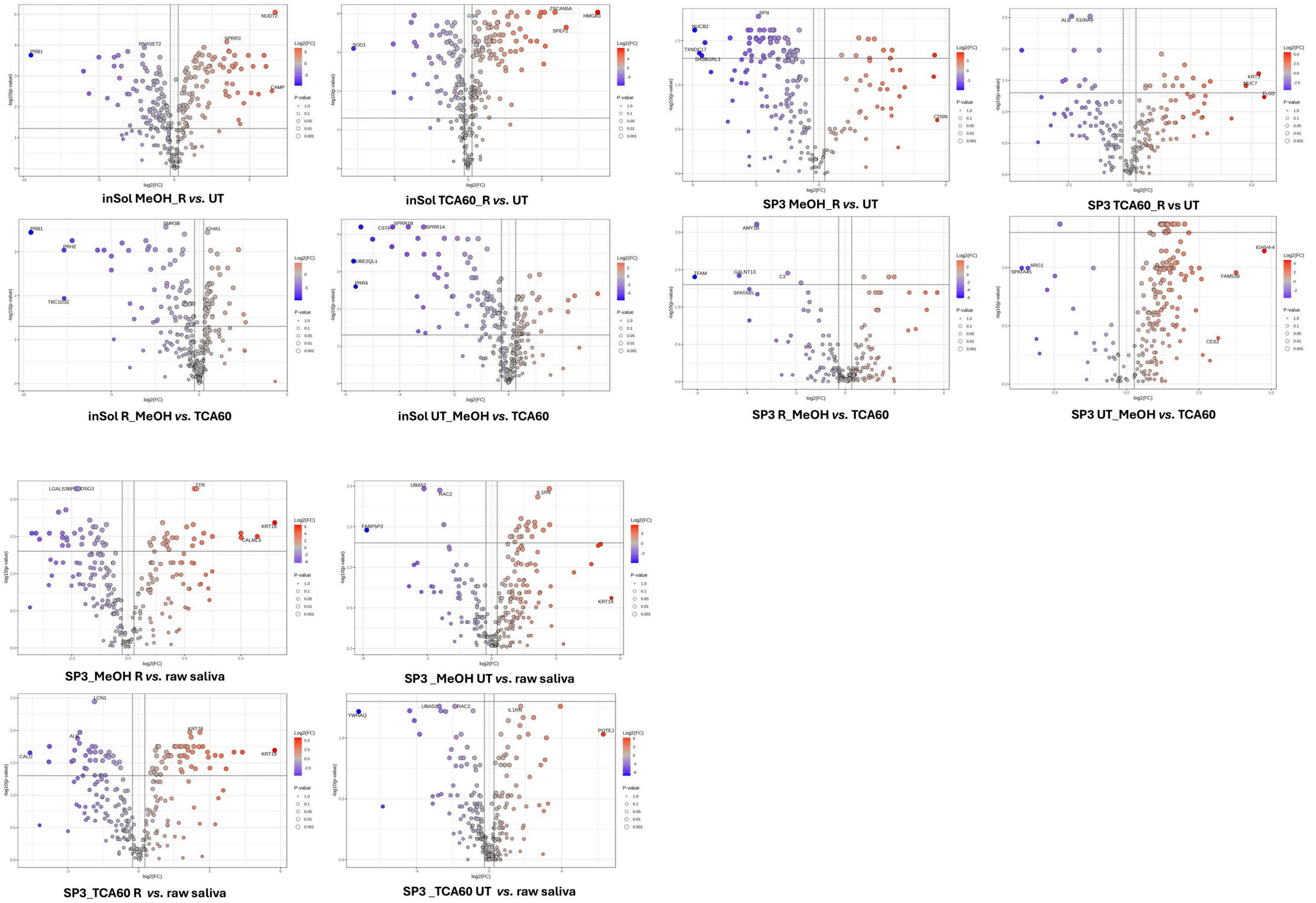

Figure S7: Volcano plots displaying the statistical p-value with the magnitude of abundance changes between each condition for in-solution and SP3 methods.
